# Light-driven bending of diarylethene mixed crystals†
†Electronic supplementary information (ESI) available: Synthetic procedures and characterization of **2a**, supplementary data, and crystallographic analysis. CCDC 1060727 and 1408475. For ESI and crystallographic data in CIF or other electronic format See DOI: 10.1039/c5sc01994j



**DOI:** 10.1039/c5sc01994j

**Published:** 2015-07-14

**Authors:** Satoko Ohshima, Masakazu Morimoto, Masahiro Irie

**Affiliations:** a Department of Chemistry and Research Center for Smart Molecules , Rikkyo University , Nishi-Ikebukuro 3-34-1 , Toshima-ku , Tokyo 171-8501 , Japan . Email: iriem@rikkyo.ac.jp

## Abstract

Mixed crystals composed of 1,2-bis(2-methyl-5-(*p*-methoxyphenyl)-3-thienyl)perfluorocyclopentene (**1a**) and 1,2-bis(5-methyl-2-(*p*-methoxyphenyl)-4-thiazolyl)perfluorocyclopentene (**2a**) were prepared, and their photochromic as well as light-driven bending performance was studied to reveal how shape changes of individual molecules cause the bending response. **1a** and **2a** molecules, having similar geometrical structures, randomly mix with each other in a single crystal. The absorption spectra of the closed-ring isomers **1b** and **2b** were, however, distinctly different. The difference of the spectra made it possible to discriminate the cycloreversion reactions of **1b** and **2b** in the mixed crystals by irradiation with 750 nm light. The bending response of the mixed crystals by the selective photoisomerization revealed that the local shape change of each molecule is additively linked to the macroscopic deformation of the crystals.

## Abstract 2

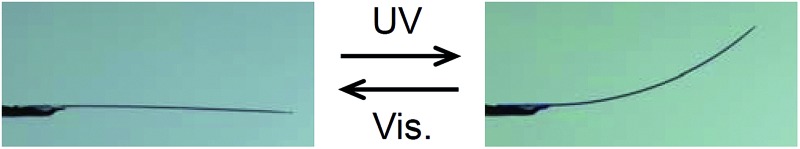
The bending response of mixed crystals by selective photoisomerization revealed that the local shape change of each molecule is additively linked to the macroscopic deformation of the crystals.

## Introduction

It is of particular interest from both scientific and technological points of view to construct molecular systems that can make mechanical motion based on the geometrical structure or shape changes of individual molecules induced by external stimuli, such as chemicals, photons or electrons (holes), and have these systems perform macroscopic mechanical work.^1^ Mechanical work means the controlled, large amplitude, or directional motion of materials. In biological systems, the molecular-scale sliding movements of actin–myosin proteins are artfully linked to the macroscopic motion of muscles.^2^ Although various types of elaborate molecular systems have been reported,^3^ an attempt to link the molecular-scale movement of these man-made devices to the macroscopic motion of materials is limited. Any certain methodology on how to rationally assemble these molecular devices into materials that can perform significant work at the macroscopic level has not yet been developed. Feringa and co-workers^4^ have developed light-driven unidirectional molecular rotary motors and doped a liquid-crystal film with the motors to amplify the molecular phenomena to the visible macroscopic movement of a glass rod. The movement was, however, too subtle to be put to practical use. On the other hand, various types of polymers,^5^ polymer brushes,^6^ amorphous microfibers,^7^ and carbon nanotubes^8^ have been reported to convert molecular phenomena into macroscopic motion of materials. The motion, however, relies not on the individual molecular behaviour but on the response of bulk materials. The photoinduced contraction of liquid-crystal elastomers^5*a*^ is attributed to an order–disorder transition of the liquid-crystal material. The photomechanical bending of amorphous microfibers made of azobenzene derivatives^7^ is related to photoinduced mass transport near the irradiated surfaces of the fibers. It is a challenge to construct molecular systems that perform macroscopic mechanical work based on photostimulated geometrical structure or shape changes of individual molecules.

In previous papers^9^ we have demonstrated that plate or rod-like diarylethene single crystals exhibit a bending action upon alternate irradiation with UV and visible light. In addition to diarylethene crystals, various other crystalline materials have been reported to exhibit such a photomechanical bending response.^10–16^ In most cases the effect is due to gradients in the extent of the photoreactions caused by the high light absorption of the crystals. The contraction or expansion of the irradiated surface of the crystals induces the bending, as observed in a bimetal. The photogenerated anisotropic strain in the crystals is considered to bring about the deformation of the crystal surface. Here, we studied whether the local shape change of each molecule additively contributes to the strain, or whether a cooperative phase transition or domain formation induced by the photoisomerization produces the strain.

## Results and discussion

### Photoisomerization in mixed crystals

We employed mixed crystals composed of 1,2-bis(2-methyl-5-(*p*-methoxyphenyl)-3-thienyl)perfluorocyclopentene^17^ (**1a**) and 1,2-bis(5-methyl-2-(*p*-methoxyphenyl)-4-thiazolyl)perfluorocyclopentene (**2a**) to reveal the contribution of individual molecules to the strain.
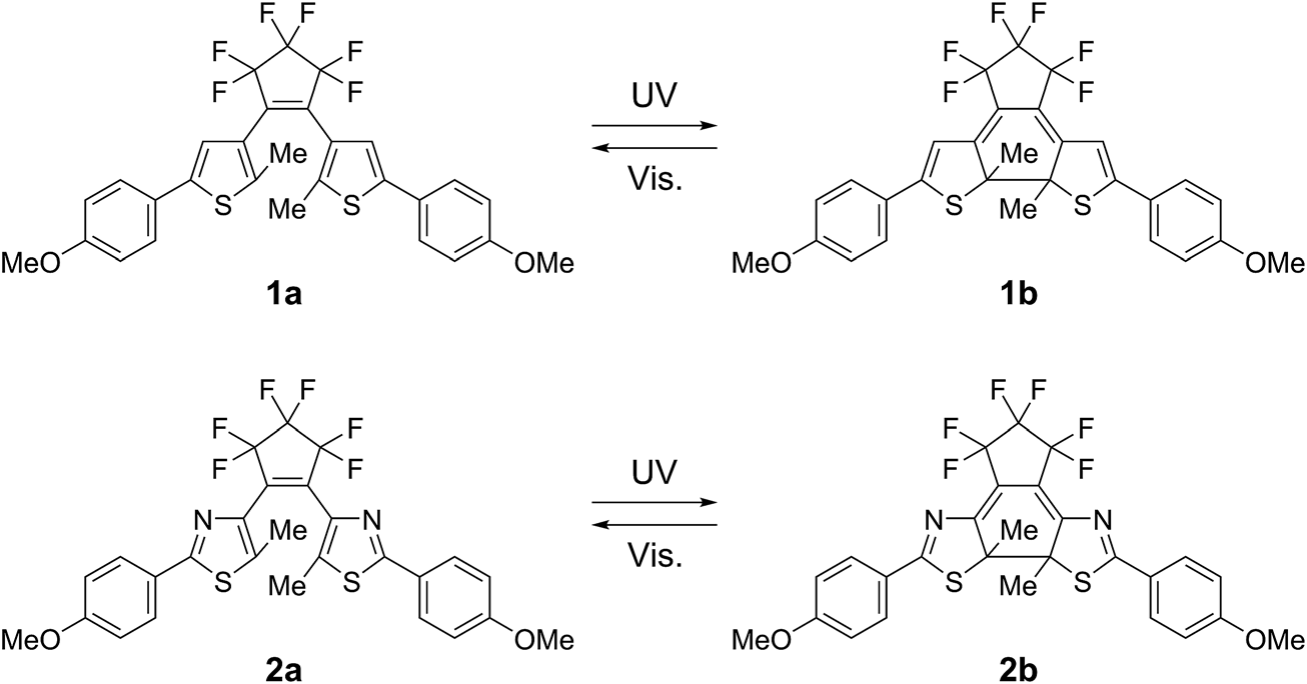


**Fig. 1:**
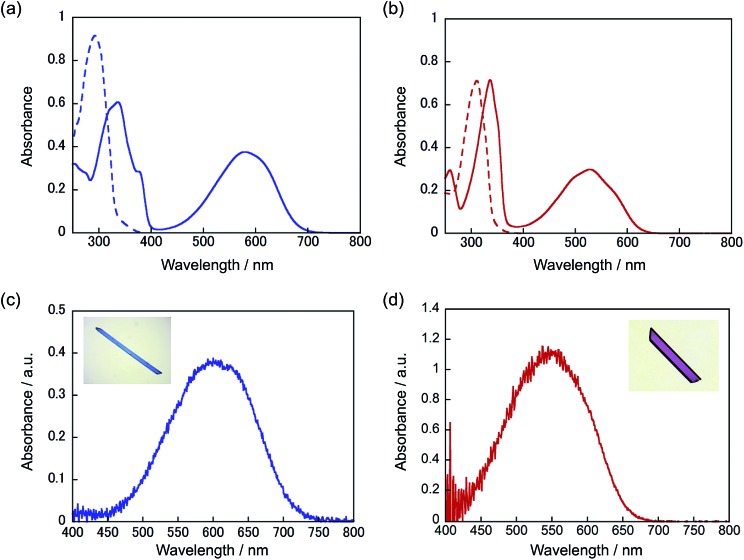
Absorption spectra changes for **1a** and **2a** upon irradiation with 365 nm light in *n*-hexane solution ((a) and (b)) and in crystal form ((c) and (d)). (a) and (b) **1a** (blue broken line, 2.2 × 10^–5^ M); **2a** (red broken line, 1.6 × 10^–5^ M); under photostationary states (blue and red solid lines). (c) and (d) Absorption spectra of the crystal of **1a** (blue solid line) and the crystal of **2a** (red solid line) after irradiation with 365 nm light. Photographs in (c) and (d) show the coloured single-component crystals **1a** and **2a** after irradiation with 365 nm light, respectively.

The **1a** and **2a** molecules have similar geometrical structures but different absorption spectra in their closed-ring isomers. Fig. 1 shows the absorption spectra changes for **1a** and **2a** upon irradiation with 365 nm light in solution and in a crystal form. In *n*-hexane solution the closed-ring isomer **1b** has an absorption maximum at 580 nm, while the maximum was observed at 535 nm in the closed-ring isomer **2b**. In crystal the maxima of the closed-ring isomers **1b** and **2b** showed bathochromic shifts to 600 nm and 550 nm, respectively.

**Fig. 2:**
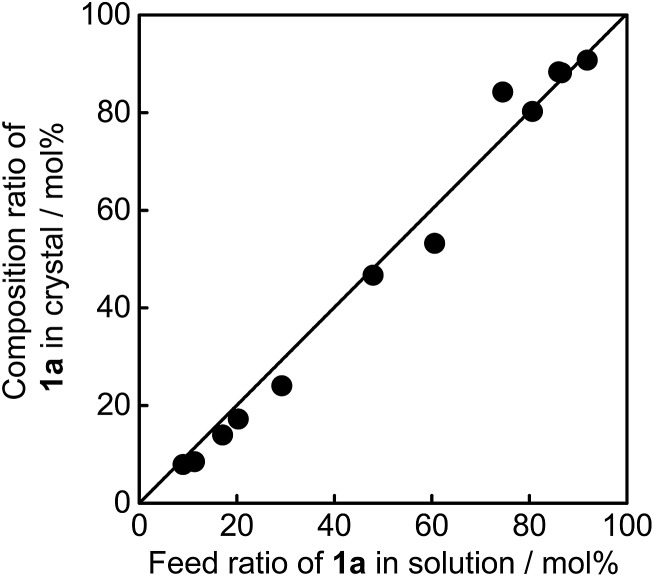
Relationship between the content of **1a** in the feed ethanol solution and the composition content of **1a** in the mixed crystal obtained. The error range in the composition content of **1a** in crystals was ±5 mol%.

These two molecules, having similar geometrical structures, readily mix with each other in a single crystal. Mixed crystals were prepared by recrystallization from ethanol. Fig. 2 shows the relationship between the content of **1a** in the feed ethanol solution and composition content of **1a** in the mixed single crystal obtained. The linear correlation between the content of **1a** in the crystals and the content of **1a** in the feed solutions indicates that these two molecules randomly mix in the single crystals. Upon irradiation with 365 nm light, the mixed crystals changed colour from colourless to violet-blue, depending on the concentration of **1a** and **2a**. The violet-blue colour disappeared upon irradiation with visible (>500 nm) light. Both single component crystals also undergo photochromic reactions upon alternate irradiation with UV and visible light. The single component crystal of **1a** turned blue upon irradiation with 365 nm light, while the crystal of **2a** turned violet. The rate of colouration was similar for each. The colour change of the mixed crystals is, therefore, ascribed to the photogenerated closed-ring isomers **1b** and **2b**. The conversion ratio from the open- to the closed-ring isomers was relatively low, less than 15%, in the crystals.^9d,f,g^

**Fig. 3:**
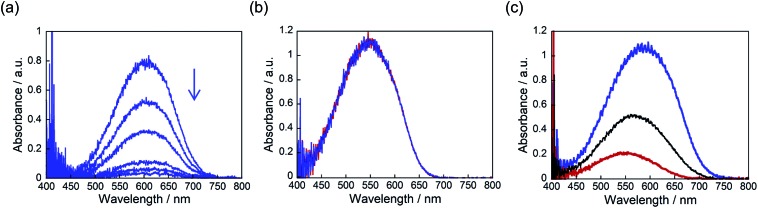
Absorption spectra changes for the coloured isomers (a) **1b** and (b) **2b** in the single-component crystals of **1a** and **2a** upon irradiation with 750 nm light. (a) The crystal of **1a** was irradiated with 365 nm light for 30 s and then bleached with 750 nm for 2 min, 4 min, 6 min, 8 min, and 10 min. (b) The crystal of **2a** was irradiated with 365 nm light for 30 s (blue line) and subsequently irradiated with 750 nm light for 10 min (red line). (c) Selective photo-bleaching of coloured isomers **1b** and **2b** in a mixed crystal (**1a**/**2a** = 60/40 mole ratio in the feed solution) upon irradiation with 750 nm light: after irradiation with 365 nm light for 30 s (blue line); after irradiation with 750 nm light for 10 min (black line) and 20 min (red line). The band at 550 nm (red line) remained even after irradiation for 30 min.

Fig. 3 shows the absorption spectra changes for the coloured closed-ring isomers **1b** and **2b** in the single component crystals of **1a** and **2a**, and in a mixed crystal upon irradiation with 750 nm (750 ± 10 nm) light. The crystals were initially coloured by irradiation with 365 nm light, and then bleached by irradiation with 750 nm light. In the single component crystals the absorption at 600 nm due to the **1b** isomer disappeared upon irradiation with 750 nm light, while the absorption at 550 nm due to **2b** isomer remained constant even after irradiation for 10 min. This result indicates that 750 nm light can convert **1b** to **1a**, but the light cannot induce the reaction from **2b** to **2a**. The cycloreversion reactions of **1b** and **2b** can be discriminated by irradiation with 750 nm light.

The selective photoisomerization was carried out for a mixed crystal (**1a**/**2a** = 60/40 mole ratio in the feed solution), as shown in Fig. 3c. Upon irradiation with 750 nm light, the strong absorption at 600 nm considerably decreased and a small absorption band at 550 nm remained. The absorption band at 550 nm was not bleached even after irradiation for 30 min. This result indicates that in the mixed crystal **1b** was selectively bleached to **1a** by irradiation with 750 nm light, while **2b** did not undergo the isomerization reaction to **2a** (see also Fig. 8b).

**Fig. 4:**
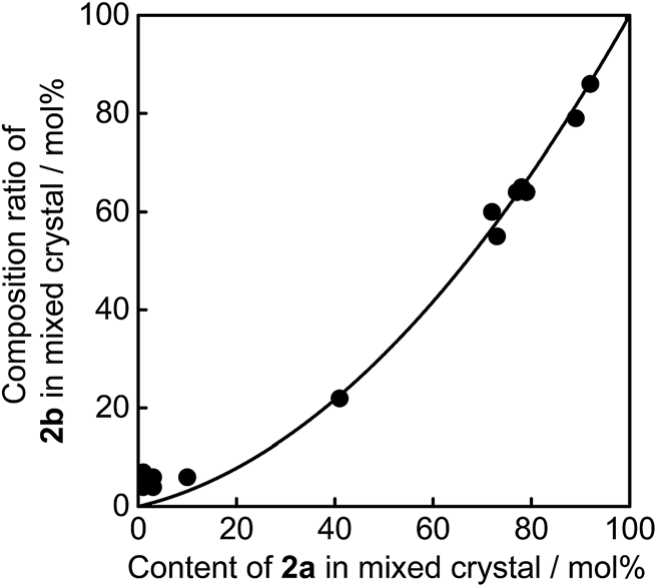
Relationship between the content of **2a** in mixed crystal before UV (365 nm) irradiation and the composition ratio of **2b** in UV irradiated crystal.

As described above, upon irradiation with 365 nm light, both **1a** and **2a** molecules undergo cyclization reactions to produce coloured isomers **1b** and **2b** in the mixed crystals. To know the ratio of **1b** and **2b** in the UV irradiated mixed crystals, the crystals were dissolved in *n*-hexane and the ratio of the coloured isomers **1b** and **2b** was measured by using high-performance liquid chromatography (HPLC). The initial contents of **1a** and **2a** in the crystals were also determined using HPLC before UV irradiation. Fig. 4 shows the relationship between the content of **2a** in the mixed crystal before UV irradiation and the composition ratio of **2b** in the UV irradiated crystal. The relationship indicates that the reaction efficiency of **1a** is superior to that of **2a**. When the initial content of **2a** was 40 mol% (**1a**/**2a** = 60/40) in the mixed crystal, the composition ratio of photogenerated **2b**, (**2b**/(**2b** + **1b**)) × 100, in the UV irradiated crystal is estimated to be 22 mol%. The higher reaction efficiency of **1a** is ascribed to the higher absorption coefficient at 365 nm and the higher cyclization quantum yield of **1a** in comparison with those of **2a**.

**Fig. 5:**
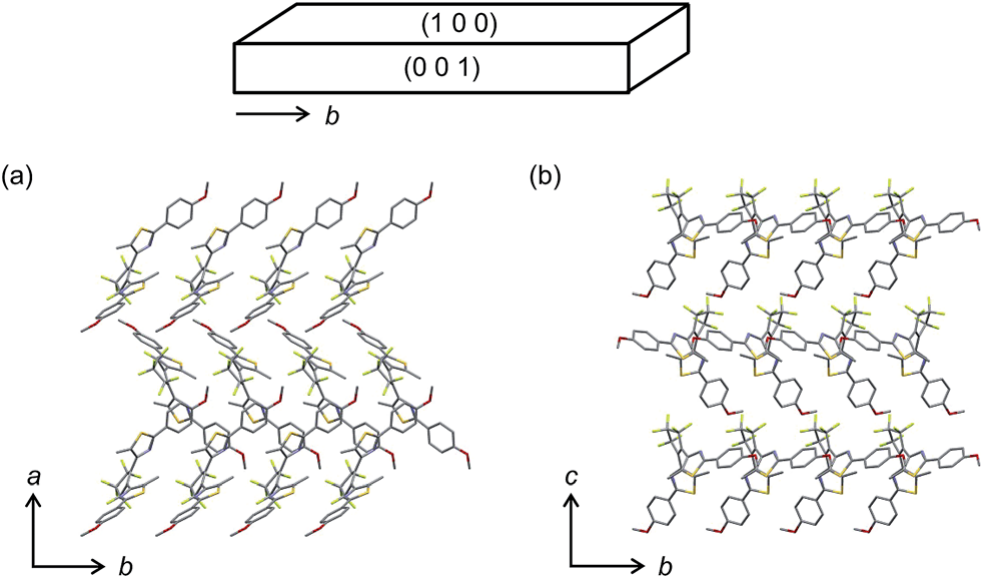
X-ray crystallographic analysis of the molecular packing of **1a** and **2a** molecules in a mixed crystal, which was prepared from an ethanol solution containing **1a** and **2a** (**1a**/**2a** = 80/20 mole ratio), viewed from the (a) (001) and (b) (100) faces.

The molecular packing and composition ratio of **1a** and **2a** in the mixed crystals were examined by X-ray crystallographic analysis. Fig. 5 shows the molecular packing of a mixed crystal, which was prepared from an ethanol solution containing **1a** and **2a** (**1a**/**2a** = 80/20 mole ratio in the feed solution), viewed from the (001) and (100) faces. The molecular packing of the mixed crystal is the same as the packing of the single component crystal of **1a**, as shown in Table S1.† The molecules are packed in an anti-parallel conformation and the distance between the reactive carbons is 0.35 nm, which is short enough for the photocyclization reaction to take place.^18^ The composition ratio of **2a** in the mixed crystal was estimated to be 24 mol% from the disorder analysis, which is slightly larger than the value (20 mol%) determined by HPLC. Roughly 1/5 of the **1a** molecules are randomly replaced with **2a** molecules in the mixed crystal.

### Light-driven bending of the mixed crystals

**Fig. 6:**
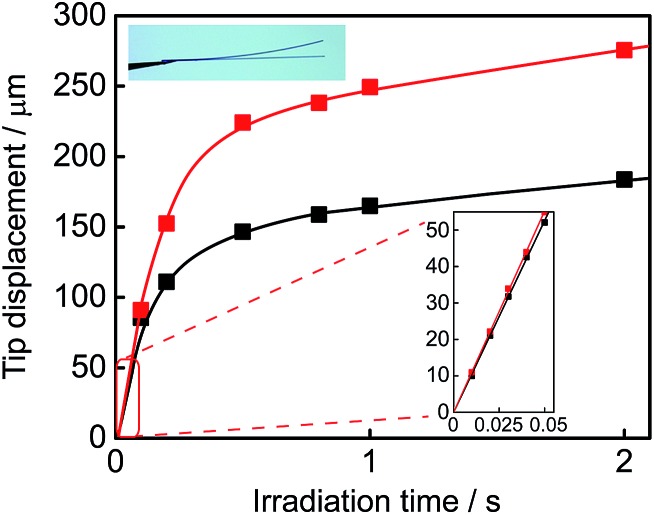
Time-dependence of the tip displacement for the crystals of **1a** (black square) and **2a** (red square) upon irradiation with 365 nm light. The sizes of the rectangular plate crystals were as follows: **1a** (length: 0.56 mm and thickness: 7.4 μm); **2a** (length: 0.48 mm and thickness: 7.9 μm). A high-speed camera (Keyence, VW-6000) was used to follow the rapid response in the initial 50 ms.

Both single component crystals **1a** and **2a** exhibited a reversible bending response upon alternate irradiation with 365 nm and visible (>500 nm) light. The crystals bent toward the UV light source. Fig. 6 shows the time-dependence of the tip displacement for crystals **1a** and **2a**. A high-speed camera (Keyence, VW-6000) was used to follow the rapid response in the initial 50 ms. Both crystals showed a very quick response without any induction period. There was no time lag in the bending response. The response time of the crystal of **2a** is faster and its final deformation is larger than those of the crystal of **1a**. To know the cause of the difference, the Young's modulus was measured by means of a manual beam-bending method (for details, see Fig. S2†). The Young's moduli of the crystals of **1a** and **2a** were determined to be 12 GPa and 6.2 GPa, respectively. The difference in the response behaviour is attributable to the smaller Young's modulus of the crystal of **2a** in comparison with that of the crystal of **1a**. The difference in the structural changes by the cyclization reactions may also contribute to the difference in the bending response.

The rectangular plate crystals could lift a metal ring, the weight of which is 1600–1900 times heavier than the crystals (Fig. S3†). A rectangular plate crystal of **1a** (0.0026 mg) with a length of 1.5 mm was fixed at the edge of a glass plate as a cantilever arm, and a metal ring (4.1 mg) was loaded on the plate. The weight was 1600 times heavier than the crystal. Upon irradiation with 365 nm light, the weight was lifted as high as 0.10 mm. The crystal performed lifting work as large as 4.0 nJ. The crystal of **2a** could lift a metal ring, the weight of which was 1900 times heavier than the crystal plate, and performed the work of 7.0 nJ. These crystals generated a strong force upon photoirradiation and performed large mechanical work.

**Fig. 7:**
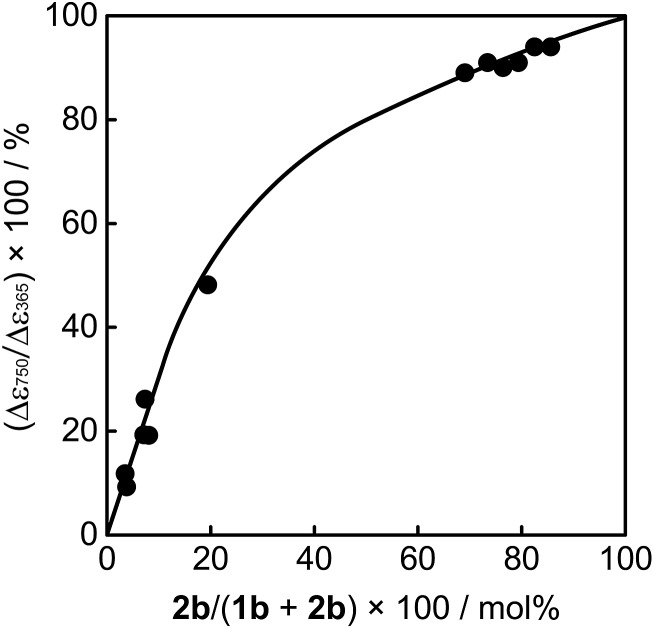
Relationship between the content of **2b** in the mixed crystal and the strain produced in the crystal by **2b**. The strain, Δ*ε*, was measured from the photo-bent shape of the crystal by using a simple cantilever model (for details, see Fig. S5†).

The mixed crystals also exhibited a reversible bending response upon photoirradiation. The bending cycle could be repeated more than 300 times upon alternate irradiation with 365 nm and visible light (Fig. S4†). As described before, the **1a** and **2a** molecules randomly mix in a single crystal and both molecules undergo photocyclization reactions upon irradiation with 365 nm light. To reveal the relative contribution of **1** and **2** molecules to the bending response, the relationship between the content of **2b** in the crystal, (**2b**/(**1b** + **2b**)) × 100, and the strain produced in the crystal by **2b** was measured, as shown in Fig. 7. The content of **2b** generated by irradiation with 365 nm in the crystals was determined from the relationship shown in Fig. 4. The strain, Δ*ε*, was measured from the photo-bent shape of the rectangular plate crystal by using a simplified bimetal model (for details, see Fig. S5†). The contraction of the surface layer, Δ*l*/*l*, was assumed to cause the bending, where *l* is the initial length of the crystal surface.
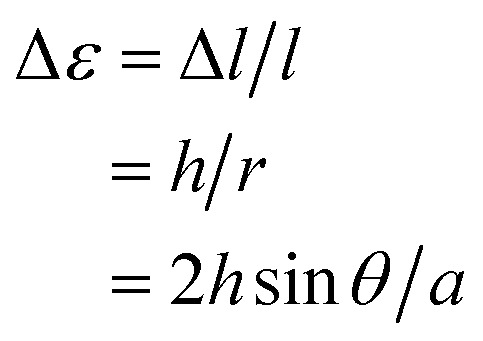


In the above equations, *r* is calculated from the distance between the tip of the crystal and the edge of the glass joint, *a*, and the bending angle, *θ*, and *h* is the thickness of the plate. *a* and *θ* can be readily measured using a digital microscope (Keyence, VHX-900). Δ*ε*_365_ is the strain produced by irradiation with 365 nm light, while Δ*ε*_750_ is the strain that remained after irradiation with 750 nm light.

**Fig. 8:**
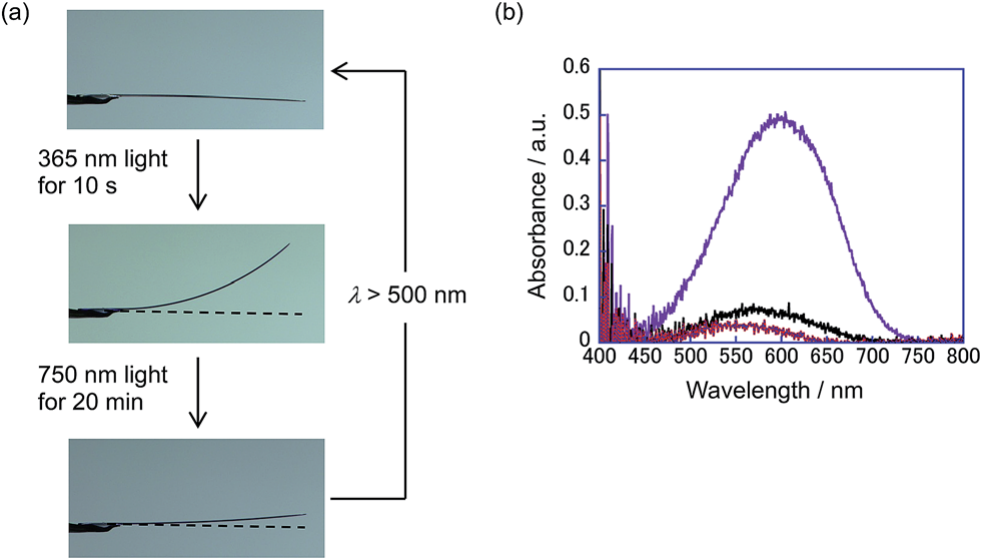
(a) Bending response of a mixed crystal (**1a**/**2a** = 80/20 mole ratio in the feed solution) and (b) absorption spectral changes of the crystal by irradiation with 365 nm and 750 nm light: after irradiation with 365 nm light for 30 s (violet line); after irradiation with 750 nm light for 10 min (black line); after irradiation with 750 nm light for 20 and 30 min (red and blue lines).

The convex upward curve shown in Fig. 7 indicates that even a small amount of **2b** molecules can bring about a relatively large bending effect. There is no threshold value of the content of **2b** molecules below which no deformation is observable. Fig. 8 shows the bending response of a mixed crystal (**1a**/**2a** = 80/20 mole ratio in the feed solution) and absorption spectra changes for the crystal. Upon irradiation with 365 nm light for 10 s, the straight crystal bends and keeps its shape after the irradiation is stopped. Then, the bent crystal was irradiated with 750 nm light for 20 min. The bending angle becomes smaller, but still the crystal kept the bent shape. After that the crystal returned back to the original straight shape by irradiation with >500 nm light. The absorption spectra changes during the above operation are shown in Fig. 8b. Upon irradiation with 750 nm light, the strong absorption around 600 nm dramatically decreases, but a small absorption remains even after prolonged irradiation. The small absorption is due to the **2b** molecules that remained in the crystal. The amount of remaining **2b** molecules is estimated to be less than 8 mol%. Even so low content of **2b** molecules induced a bending as large as 15% of the initial bending upon irradiation with 365 nm light.

After irradiation with 750 nm light, the remaining **2b** molecules are considered to be distributed randomly in the crystal, possibly in isolation. The random distribution of the small numbers of **2b** molecules in the crystal indicates that domain formation (or separation) between reacted and unreacted molecules is unlikely to take place. A cooperative phase transition is also not anticipated to occur at such a low concentration of **2b** molecules. In addition, the bending response at extremely low temperature, below 5 K, as observed in previous papers^9f,g^ explicitly excludes the possibility that the phase transition plays a role to produce the strain.

**Fig. 9:**
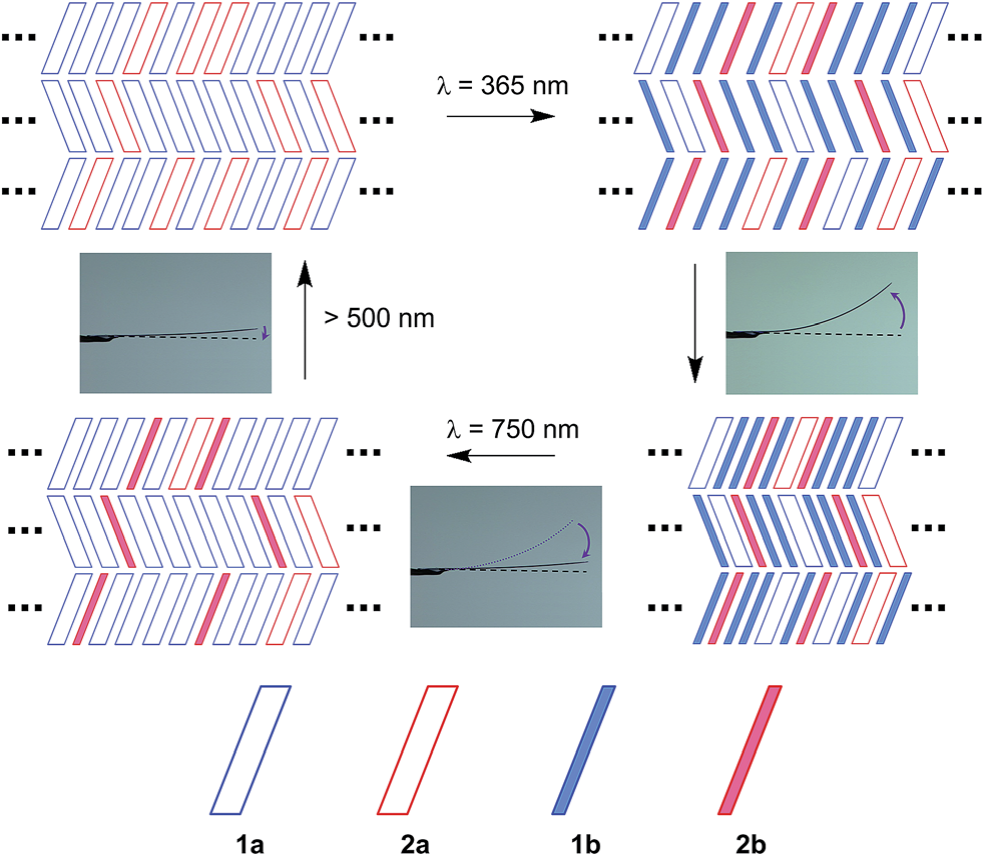
Schematic illustration of the reactions in the crystal upon irradiation with 365 nm and 750 nm light and the bending response of the crystal.

Fig. 9 shows a schematic illustration of the reactions in the present crystals upon irradiation with 365 nm and 750 nm light. In the surface layers, molecules are packed as shown in Fig. 5. Upon 365 nm light irradiation, the molecules in the surface layers undergo cyclization reactions. Both **1a** and **2a** molecules change their shape during the reactions (Fig. S6†). The height of the triangle shape of the molecules increases and the width decreases. At the same time, the thickness is reduced due to the rotation of thiophene or thiazole rings. The anisotropic shape changes cause the contraction. Cofacial packing of the layers of the thin **1b** and **2b** molecules along the *b*-axis allows the molecules to be stacked one-by-one, resulting in a contraction along the *b*-axis, as reported in previous papers.^9^ Upon 750 nm light irradiation, the **1b** molecules convert back to the original thick **1a** molecules, while the **2b** molecules remain unreacted and isolated in the molecular layers. Although the reversion of **1b** to **1a** considerably decreases the bending angle, the remaining **2b** molecules still induce the bending to some extent. Even isolated **2b** molecules can cause the bending of the crystal. This result indicates that the shape change of each molecule is responsible for the macroscopic deformation of the plate crystal.

## Conclusions

In conclusion, the bending response of the mixed crystals by selective photoisomerization after irradiation with 750 nm light revealed that the local shape change of each molecule is additively linked to the macroscopic deformation of the crystals. Fabrication of single crystalline assemblies is a promising approach to integrate photoswitching molecules into macroscopic materials that perform significant mechanical work.

## Experimental

**1a** was synthesized by the method reported previously.^17^ The synthesis of **2a** is described in the ESI.† Commercially available reagents and solvents for the syntheses were used without further purification. Solvents for spectral measurements were of spectroscopic grade. The single crystals were prepared by recrystallization from ethanol solutions.

The ^1^H NMR spectra were measured with an NMR spectrometer (Bruker, Avance 400). Tetramethylsilane was used as an internal standard. The mass spectra were measured with a gas chromatography-mass spectrometer (Shimadzu, GCMS-QP2010Plus). Elemental analysis was performed with an elemental micro analysis system (Elementar, Vario MICRO Cube). The absorption spectra of solutions were measured with an absorption spectrophotometer (Hitachi, U-4100). The absorption spectra of single crystals were measured using a Leica DMLP polarizing microscope connected with a Hamamatsu PMA-11 photodetector. Photoirradiation was carried out using a UV-LED irradiation system (Keyence, UV-400 with UV-50H; wavelength: 365 nm, 10–40 mW cm^–2^) or a 300 W xenon lamp (Asahi spectra, MAX-302). The wavelength of the light was selected using band-pass or cut-off optical filters. Composition ratios of the crystals were analyzed using an HPLC system (Hitachi L-2130 pump system, L-2420 detector, Wakosil 5SIL (*φ*4.6 mm × 250 mm), and *n*-hexane : ethyl acetate = 80 : 20, 1.0 mL min^–1^). The photographs of the crystals were recorded with a digital microscope (Keyence, VHX-900) and a high-speed camera (Keyence, VW-6000).

The X-ray crystallographic analysis was performed with a CCD-based X-ray diffractometer (Bruker AXS, SMART APEX2 Ultra-Cu) with Cu K_α_ radiation (*λ* = 1.54178 Å) for the crystal of **1a**/**2a** and another X-ray diffractometer (Bruker AXS, SMART APEX) with Mo K_α_ radiation (*λ* = 0.71073 Å) for the crystal of **2a**. The crystals were cooled by a low temperature controller (Japan Thermal Engineering, TC-190CP-CS-K). The diffraction frames were integrated with the Bruker SAINT software package. The cell constants were determined by global refinement. The structures were solved by a direct method using the SHELXS-97 and SHELXS-90 programs for the crystals of **1a**/**2a** and **2a**, respectively. The solved structures were refined by a full-matrix least-squares method using the SHELXL-2014 and SHELXL-97 programs for the crystals of **1a**/**2a** and **2a**, respectively. The positions of all the hydrogen atoms were calculated geometrically and refined by the riding model.
