# Supplementary material for: Light-driven bending of diarylethene mixed crystals
Source: Chem Sci. 2015 Jul 14;6(10):5746–52. doi: 10.1039/c5sc01994j (PMC5515063; doi:10.1039/c5sc01994j)
Supplement: Supplementary file 1 [file SC-006-C5SC01994J-s001.pdf]

Electronic Supplementary Material (ESI) for Chemical Science.
This journal is © The Royal Society of Chemistry 2015

Electronic Supplementary Information

Light-driven bending of diarylethene mixed crystals

Satoko Ohshima,[a] Masakazu Morimoto[a] and Masahiro Irie*[a]

[a] *Department of Chemistry and Research Center for Smart Molecules, Rikkyo University, Nishi-Ikebukuro 3-34-1, Toshima-ku, Tokyo 171-8501, Japan. E-mail:iriem@rikkyo.ac.jp*

Scheme S1 Synthesis of **2a**.

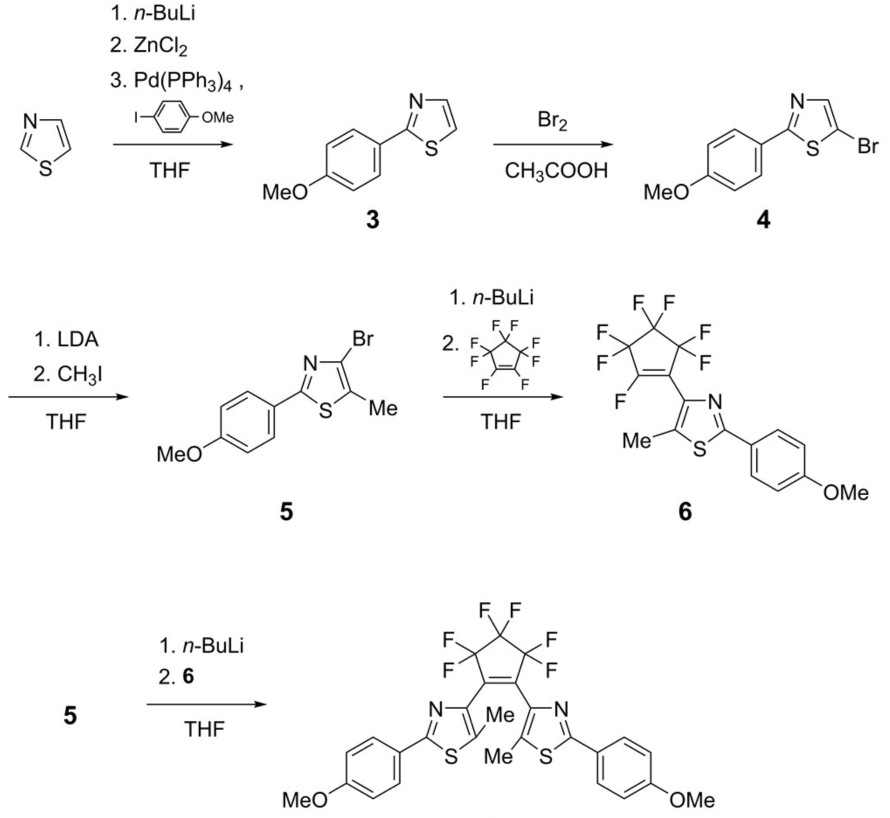

2-(*p*-Methoxyphenyl)thiazole (3)

To a dry THF solution (150 mL) containing thiazole (5.4 g, 63 mmol) was slowly added 1.6 M *n*-BuLi hexane solution (84.0 mL, 134 mmol) at −78°C under a nitrogen atmosphere and the solution was stirred for 15 min. $ZnCl_2$ (30.0 g, 220 mmol) was added and the mixture was stirred for 15 min. After warming to room temperature, 4-iodoanisole (18 g, 77 mmol) and $Pd(PPh_3)_4$ (4.0 g, 3.5 mmol) was added and the mixture was refluxed at 70°C for 3 h. The reaction mixture was extracted with ethyl acetate. The organic layer was dried over $MgSO_4$, filtrated, and concentrated. The residue was purified by silica gel column chromatography (hexane : chloroform = 1 : 3) to afford **3** as a yellow oil (9.1 g, 75%). ^{1}H NMR: δ_H (400MHz; $CDCl_3$; Me_4Si) 3.86 (3H, s), 6.96 (2H, d, J = 8.8 Hz), 7.26 (1H, d, J = 3.2 Hz), 7.81 (1H, d, J = 3.2 Hz), 7.91 (2H, d, J = 8.8 Hz). MS (EI): m/z 191 (M^+).

5-Bromo-2-(*p*-methoxyphenyl)thiazole (4)

To an acetic acid solution (80 mL) containing **3** (5.6 g, 29 mmol) and sodium acetate (4.1 g, 50 mmol) was slowly added an acetic acid solution (10 mL) of bromine (1.5 mL, 29 mmol) and the solution was stirred overnight. The reaction mixture was neutralized with aqueous NaOH and then extracted with ethyl acetate. The organic layer was dried over $MgSO_4$, filtrated, and concentrated. The residue was purified by silica gel column chromatography

(hexane : ethyl acetate = 9 : 1) to afford **4** as white powders (5.4 g, 69%). ^{1}H NMR: δ_H (400MHz; $CDCl_3$; Me_4Si) 3.86 (3H, s), 6.95 (2H, d, *J* = 8.8 Hz), 7.67 (1H, s), 7.80 (2H, d, *J* = 8.8 Hz). MS (EI): *m*/*z* 271 (M^++2), 269 (M^+).

4-Bromo-2-(*p*-methoxyphenyl)-5-methylthiazole (5)

To a dry THF solution (50 mL) containing diisopropyl amine (1.4 g, 14 mmol) was slowly added 1.6 M *n*-BuLi hexane solution (9.0 mL, 14 mmol) dissolved in 10 mL of dry diethyl ether at −78°C under a nitrogen atmosphere and the solution was stirred for 30 min. Then, a dry THF solution of **4** (3.3 g, 12 mmol) was added and the solution was stirred for 50 min at the low temperature. Iodomethane (1.0 mL, 16 mmol) was slowly added and the stirring was continued for 1 h at −40 °C. After warming to room temperature, the reaction was stopped by the addition of water. The reaction mixture was neutralized with HCl and then extracted with diethyl ether. The organic layer was dried over $MgSO_4$, filtrated, and concentrated. The residue was purified by column chromatography on silica gel (hexane : chloroform = 1 : 3) to afford **5** (2.7 g, 68%). ^{1}H NMR: δ_H (400MHz; $CDCl_3$; Me_4Si) 2.42 (3H, s), 3.85 (3H, s), 6.93 (2H, d, *J* = 8.8 Hz), 7.81 (2H, d, *J* = 8.8 Hz). MS (EI): *m*/*z* 285 (M^++2), 283 (M^+).

1-(5-Methyl-2-(*p*-methoxyphenyl)-4-thiazolyl)perfluorocyclopentene (6)

To a dry THF solution (80 mL) containing **5** (1.1 g, 3.9 mmol) was slowly added 1.6 M *n*-BuLi hexane solution (4.0 mL, 6.4 mmol) at −78 °C under a nitrogen atmosphere and the solution was stirred for 20 min. Octafluorocyclopentene (1.0 mL, 7.5 mmol) was added to the reaction mixture and the mixture was stirred overnight at the low temperature. After warming to room temperature, the reaction was stopped by the addition of ethanol. The reaction mixture was extracted with diethyl ether. The organic layer was dried over $MgSO_4$, filtrated, and concentrated. The residue was purified by column chromatography on silica gel (hexane : chloroform = 1 : 3) afford **6** (0.51 g, 33%). ^{1}H NMR: δ_H (400MHz; $CDCl_3$; Me_4Si) 2.51 (3H, d, *J* = 2.8 Hz), 3.86 (3H, s), 6.95 (2H, d, *J* = 8.8 Hz), 7.84 (2H, d, *J* = 8.8 Hz). MS (EI): *m*/*z* 397 (M^+).

1,2-Bis(5-methyl-2-(*p*-methoxyphenyl)-4-thiazolyl)perfluorocyclopentene (2a)

To a dry THF solution (20 mL) containing **5** (1.0 g, 3.5 mmol) was slowly added 1.6 M *n*-BuLi hexane solution (2.5 mL, 4.0 mmol) at −78 °C under a nitrogen atmosphere and the solution was stirred for 30 min. **6** (1.4 g, 3.5 mmol) dissolved in 10 mL of dry THF was slowly added to the reaction mixture and the mixture was stirred for 4 h. After warming to room temperature, the reaction was stopped by the addition of methanol. The reaction mixture was extracted with diethyl ether. The organic layer was dried over $MgSO_4$, filtrated, and concentrated. The residue was purified by column chromatography on silica gel (hexane : chloroform = 1 : 3) and recrystallization from hexane to afford **2a** as a white powder (0.27 g,

13%). ^{1}H NMR: δ_H (400MHz; $CDCl_3$; Me_4Si) 2.07 (6H, s), 3.85 (6H, s), 6.93 (4H, d, *J* = 8.8 Hz), 7.81 (4H, d, *J* = 8.8 Hz). MS (EI): *m*/*z* 582 (M^+). Anal. Calc. for $C_{27}H_{20}F_6N_2O_2S_2$: C, 55.67; H, 3.46; N, 4.81; S, 11.01. Found: C, 55.77; H, 3.57; N, 4.78; S, 11.01%.

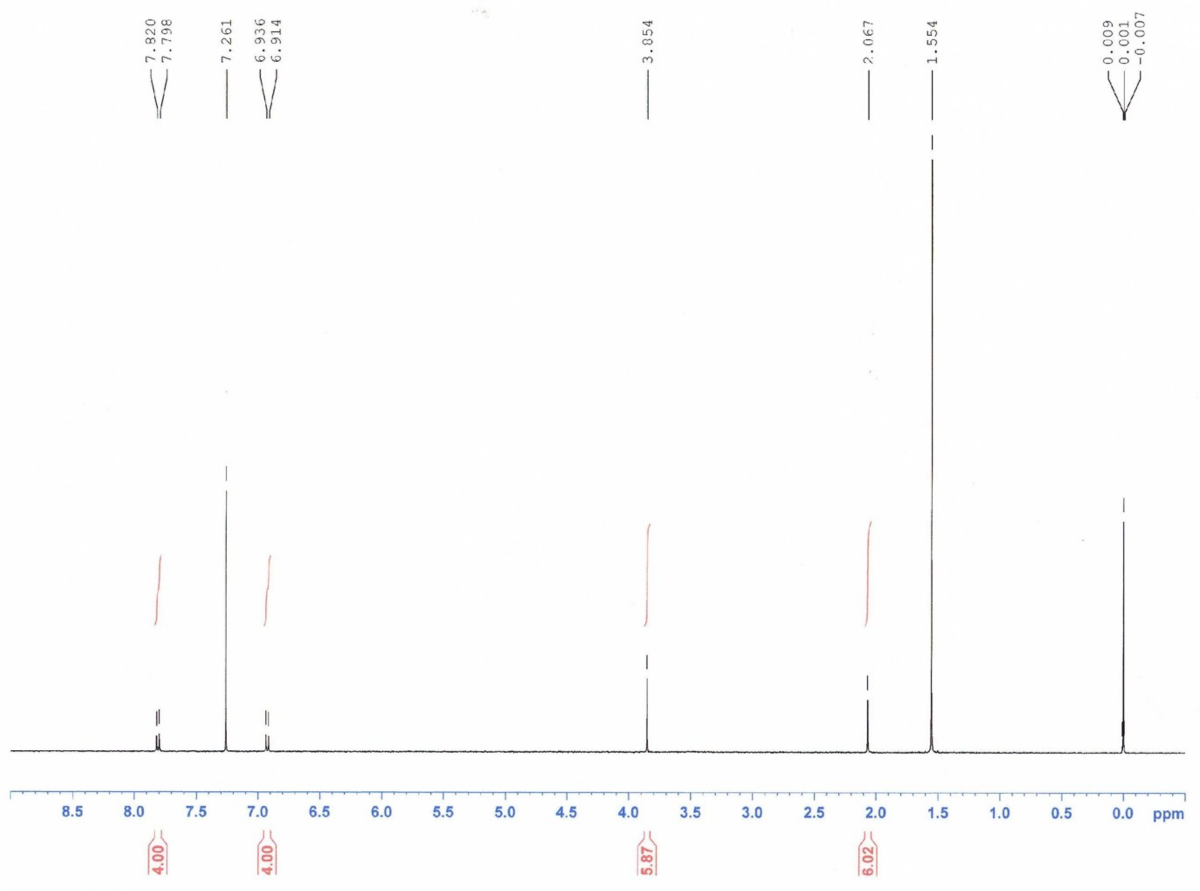

Fig. S1 ^{1}H NMR spectrum (400MHz, $CDCl_3$, Me_4Si) of **2a**.

Table S1 Crystal data for **1a**, **1a/2a**, and **2a**.

	**1a**	**1a/2a** (80/20 in feed solution)	**2a**
formula	$C_{29}H_{22}F_6O_2S_2$	$(C_{29}H_{22}F_6O_2S_2)_{0.76}\cdot(C_{27}H_{20}F_6N_2O_2S_2)_{0.24}$	$C_{27}H_{20}F_6N_2O_2S_2$
formula weight	580.59	581.06	582.57
T / K	293(2)	93(2)	93(2)
crystal system	monoclinic	monoclinic	triclinic
space group	$P2_1/c$	$P2_1/c$	$P\bar{1}$
a / Å	18.727(4)	18.2529(5)	15.141(4)
b / Å	6.6427(13)	6.5741(2)	18.267(5)
c / Å	21.459(4)	21.2658(6)	19.761(5)
α / °	90	90	72.453(5)
β / °	101.035(3)	100.9980(14)	86.674(6)
γ / °	90	90	77.582(6)
V / Å^3	2620.1(9)	2504.95(13)	5089(2)
Z	4	4	8
R_1 ($I > 2\sigma(I)$)	0.0389	0.0384	0.0770
wR_2 ($I > 2\sigma(I)$)	0.0878	0.1018	0.1389
R_1 (all data)	0.0810	0.0426	0.1212
wR_2 (all data)	0.1019	0.1055	0.1561
CCDC No.	185945	1060727	1408475

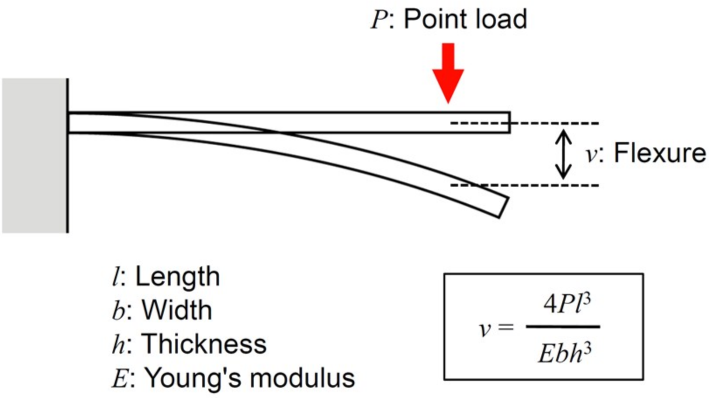

Fig. S2 Schematic illustration of an experimental setup and an equation used for the measurement of Young's modulus by a manual beam-bending method. The beam used was prepared by fixing one of edges of a crystal on a glass plate with glue. A static point load was applied by hanging a weight on the beam and the flexure of the beam was measured. The Young's modulus was calculated using the above equation.

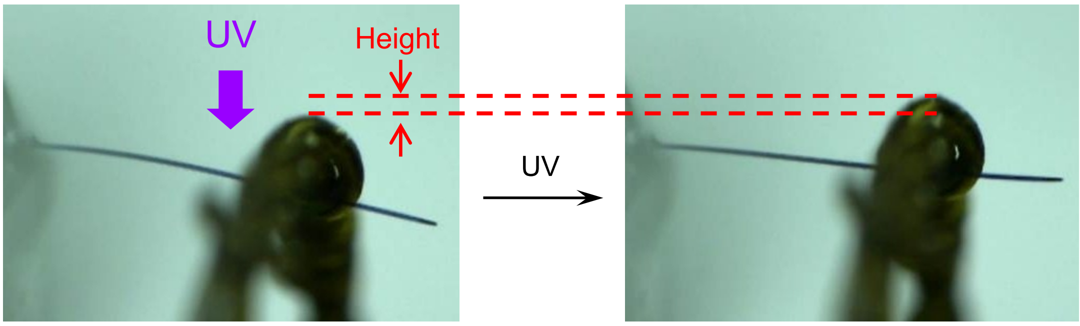

Crystal **1a**

Weight of crystal: 0.0026 mg

Length of crystal: 1.5 mm

Weight of metal: 4.1 mg

Height: 0.100 mm

Work performed: 4.0 nJ

Crystal **2a**

Weight of crystal: 0.0076 mg

Length of crystal: 1.5 mm

Weight of metal: 14.0 mg

Height: 0.050 mm

Work performed: 7.0 nJ

Fig. S3 Mechanical works performed by molecular-crystal cantilevers of **1a** and **2a** upon UV irradiation.

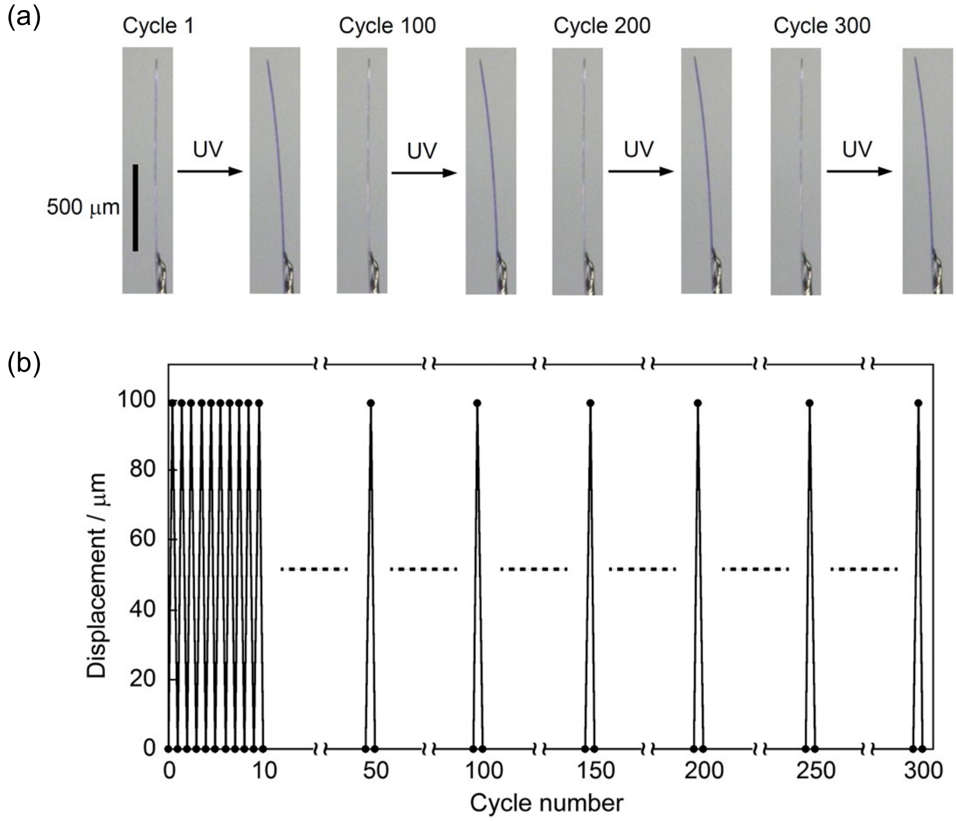

Fig. S4 Reversible bending of a two-component mixed crystal of **1a** and **2a** (**1a**/**2a** = 80/20 mole ratio in feed solution) upon alternate irradiation with UV (λ = 365 nm) and visible (λ > 450 nm) light. (a) Photographs of the crystal in the 1st, 100th, 200th and 300th bending cycles. (b) Tip displacement of the crystal during the reversible bending.

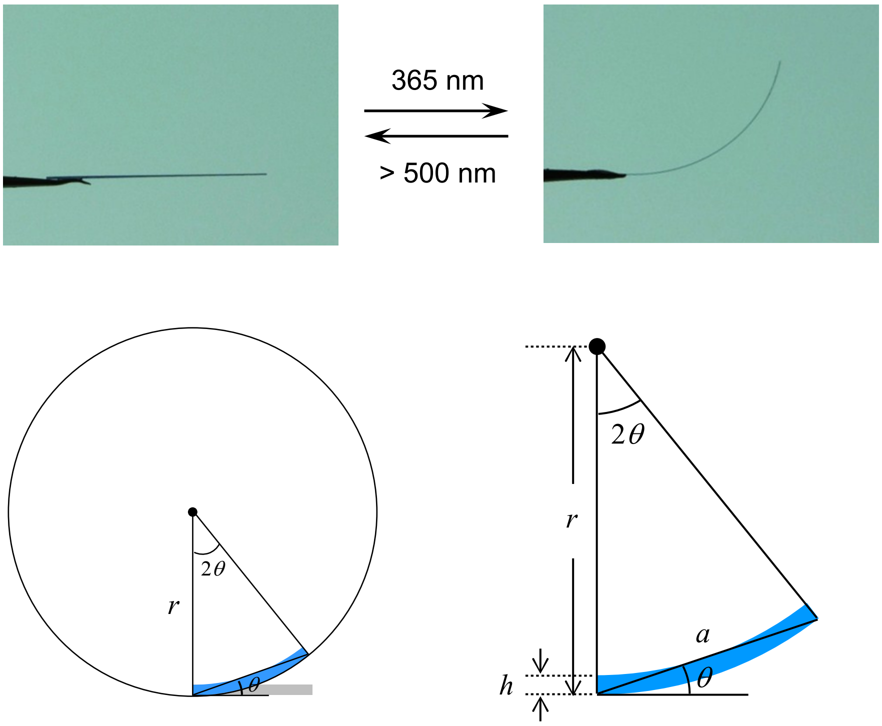

l: Length of outer arc (length of crystal)

l': Length of inner arc (length of irradiated surface)

r: Radius of outer arc (radius of curvature of bent crystal)

2θ: Central angle of arcs

a: Length of chord (distance between two ends of bent crystal)

h: Thickness of crystal

$$r = a / 2\sin\theta$$

$$\begin{aligned}\Delta\varepsilon &= \Delta l / l \\ &= (l - l') / l \\ &= [\,2\theta r - 2\theta(r - h)\,] / 2\theta r \\ &= h / r \\ &= 2h \sin\theta / a\end{aligned}$$

Fig. S5 Schematic illustration and equations for the calculation of the strain (Δε) produced by photoirradiation. In this simplified bimetal model the length of the irradiated surface was assumed to contract from l to l', while the length of the other surface was assumed to keep the initial length l even after photoirradiation.

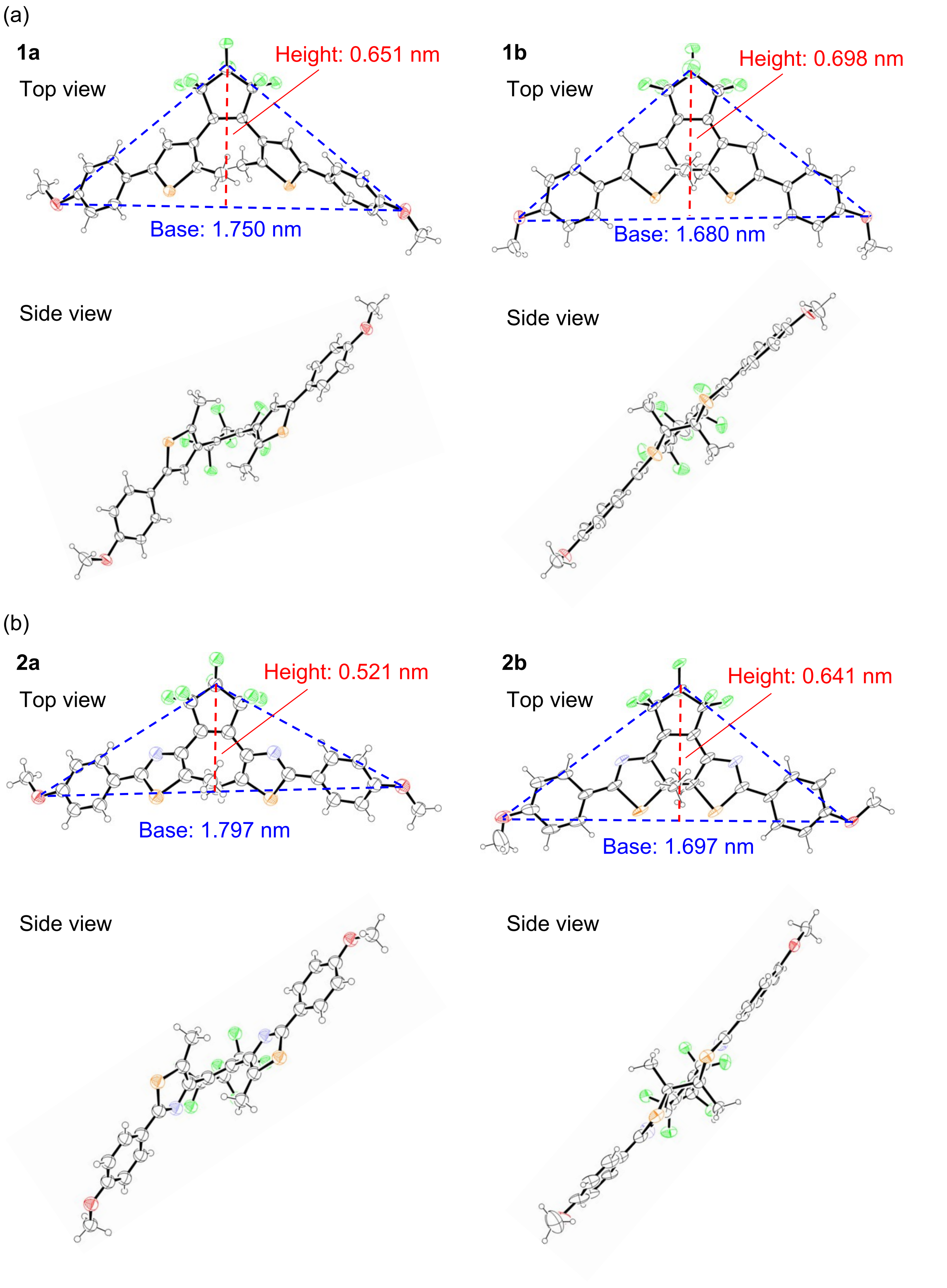

Fig. S6 Geometrical structure changes of (a) **1a** and (b) **2a** upon cyclization reactions. The molecular structures were determined by X-ray crystallographic analysis of single crystals of isolated open- and closed-ring isomers.
